# Conflicts of interest: call for new editorial policies in European national journals

**DOI:** 10.1007/s12471-012-0287-5

**Published:** 2012-05-11

**Authors:** E. E. van der Wall

**Affiliations:** 1 Interuniversity Cardiology Institute of the Netherlands – Netherlands Heart Institute, Utrecht, the Netherlands; 2 Department of Cardiology, Leiden University Medical Center, PO Box 9600, Leiden, the Netherlands

During the past decade, disclosure of conflicts of interest (COIs) has been considered the key to ensure the credibility of the scientific process [1, 2]. Biases in design, analysis, and interpretation of studies may rise when authors or sponsors have entrusted interests. Therefore, COIs should be made clear to the readers to facilitate their own judgement and interpretation of their relevance and potential implications. Authors are responsible for fully disclosing potential COIs. Failure to do so will upset the confidence of the public, health professionals and scientists in the peer-reviewed medical literature [3]. Efforts to improve transparency and protect the integrity of research, including specific recommendations and guidelines to disclose COIs, have been proposed by many organisations [4, 5]. However, ensuring adequate reporting of all sources of financial support is becoming increasingly challenging for editors as a result of the growing complexity of funding mechanisms. Furthermore, journals have different policies about COI disclosure which can cause confusion as the same author may report different information in different journals which, in turn, might jeopardise the confidence of the readers. In addition, publication of large industry-supported trials may generate many citations and journal income through reprint sales and thereby be a source of COIs for journals.

To illustrate this, Andres Lundh et al. [6] from Copenhagen, Denmark, evaluated six major medical journals (Annals of Internal Medicine, Archives of Internal Medicine, BMJ, JAMA, The Lancet, and New England Journal of Medicine [NEJM]) in order to investigate industry-supported trials’ influence on journal impact factors and revenue. The proportion of trials with sole industry support varied between journals from 7 % in BMJ to 32 % in NEJM in 2005–2006. Industry-supported trials were more frequently cited than trials with other types of support, and omitting them from the impact factor calculation decreased journal impact factors. The decrease varied considerably between journals, with 1 % for BMJ to 15 % for NEJM in 2007. For the two journals disclosing data, income from the sales of reprints contributed to 3 % and 41 % of the total income for BMJ and The Lancet in 2005–2006 (NEJM and JAMA did not respond!). The authors concluded that publication of industry-supported trials was associated with an increase in journal impact factors. It was suggested that journals disclose financial information in the same way that they require it from their authors, so that readers can assess the potential effect of different types of papers on journals’ revenue and impact.

According to the International Committee of Medical Journal Editors (ICMJE), COIs exist when an author (or the author’s institution), reviewer, or editor has financial or personal relationships that inappropriately influence (bias) his or her actions. Four main areas can be discerned: 1) authors’ associations with entities that supported the submitted manuscript, 2) associations with commercial entities with potential interest in the general area of the manuscript, 3) financial association of their spouse (!) and children, and 4) finally, non-financial associations potentially relevant to the submitted manuscript. To prevent ambiguity, authors should be explicitly asked to state whether COIs exist or do not exist. Editors should publish this information if they believe that it is important in judging the manuscript. To overcome these problems, the ICMJE proposed the use of a common vehicle to report COIs and, in October 2009, launched an electronic ‘uniform’ format for COI disclosure [7]. The reporting form has been made available at www.icmje.org/coi_disclosure.pdf.

Several years ago the European Society of Cardiology (ESC) launched the Editors’ Network with the purpose to promote the dissemination and implementation of high-quality editorial standards among ESC National Societies Cardiovascular Journals (NSCJ) [8, 9]. To determine the status of COIs and disclosure requirements among ESC NSCJ, a web-based, comprehensive, structured, and standardised questionnaire was specifically devised and sent out in June 2010. A total of 46 national journals answered the survey. Of these, 35 belong to the ESC NSCJ and 11 to journals of Affiliated Cardiac Societies. This represents a response of 83 % (35/42) of NSCJ and 58 % (11/19) for Affiliated Cardiac Societies.

In the current issue of the Netherlands Heart Journal the results of the survey are presented [10]. These data will be more or less simultaneously published this year in all 46 national journals [11]. The most important results are the following. Only 18 % of the journals have a specific policy on editors’ COIs, 25 % of the journals have policies for reviewers’ COIs, and 44 % have a specific policy on authors’ COIs. In most cases, emphasis was only made on financial COIs and on COIs directly related to the submitted work. Few journals provided definitions or examples of COIs. In most cases where COIs were requested, this policy affected all kinds of submitted articles. Written attestation by the authors was widely requested. Policies to deal with authors who fail to disclose COIs were hardly present. In most journals, the editors decided when authors’ COIs should be published but, in some journals, this information was systematically published. In more than half of the journals (54 %), reviewers were asked to decline the invitation to review if potential COIs existed. Only one-third of the editors were familiar with the new ‘Uniform Disclosure Form’ ICMJE initiative when they received the survey invitation. However, 90 % of the editors considered the ICMJE COI proposal of potential value to their particular journals and most of them declared that they were willing to implement it within a relatively short period of time.

To summarise, this survey reports the issue of ESC NSCJ policies on COI and disclosure requirements from a global and didactic perspective and provides new insights into current policies and practices among ESC NSCJ. It clearly confirms that this topic is only poorly and not uniformly dealt with by most of the national journals. In the near future, progressive steps should be taken to guarantee a systematic approach to these COI-related editorial issues. Fortunately, the ESC has recently defined a general policy for COIs [12]. In this document recommendations are given on how to minimise bias in scientific communications and continuing medical education (CME) and how to ensure proper ethical standards and transparency in relations between the medical profession and industry. It goes without saying that our journal will implement these recommendations in its editorial policies!
